# Bioabsorbable polymer optical waveguides for deep-tissue photomedicine

**DOI:** 10.1038/ncomms10374

**Published:** 2016-01-19

**Authors:** Sedat Nizamoglu, Malte C. Gather, Matjaž Humar, Myunghwan Choi, Seonghoon Kim, Ki Su Kim, Sei Kwang Hahn, Giuliano Scarcelli, Mark Randolph, Robert W. Redmond, Seok Hyun Yun

**Affiliations:** 1 Wellman Center for Photomedicine, Harvard Medical School, Massachusetts General Hospital, 65 Landsdowne Street UP-5, Cambridge, Massachusetts 02139, USA; 2 Department of Electrical and Electronics Engineering, Koc University, Istanbul 34450, Turkey; 3 SUPA, School of Physics and Astronomy, University of St Andrews, St Andrews KY16 9SS, UK; 4 Condensed Matter Department, J. Stefan Institute, Jamova 39, SI-1000 Ljubljana, Slovenia; 5 Global Biomedical Engineering, Sungkyunkwan University, Center for Neuroscience and Imaging Research, Institute for Basic Science, 2066, Seoburo, Jangan, Suwon, Gyeonggi 440-746, Korea; 6 Graduate School of Nanoscience and Technology, Korea Advanced Institute of Science and Technology, 291 Daehakro, Yuseong, Daejeon 305-701, Korea; 7 Department of Materials Science and Engineering, Pohang University of Science and Technology, San 31, Hyoja-dong, Nam, Pohang, Kyungbuk 790-784, Korea; 8 Department of Bioengineering, University of Maryland College Park, College Park,Maryland 20742, USA; 9 Division of Plastic Surgery and Harvard Medical School, Massachusetts General Hospital, 40 Blossom Street, Boston, Massachusetts 02114, USA; 10 Harvard–MIT Health Sciences and Technology, 77 Massachusetts Avenue, Cambridge, Massachusetts 02139, USA

## Abstract

Advances in photonics have stimulated significant progress in medicine, with many techniques now in routine clinical use. However, the finite depth of light penetration in tissue is a serious constraint to clinical utility. Here we show implantable light-delivery devices made of bio-derived or biocompatible, and biodegradable polymers. In contrast to conventional optical fibres, which must be removed from the body soon after use, the biodegradable and biocompatible waveguides may be used for long-term light delivery and need not be removed as they are gradually resorbed by the tissue. As proof of concept, we demonstrate this paradigm-shifting approach for photochemical tissue bonding (PTB). Using comb-shaped planar waveguides, we achieve a full thickness (>10 mm) wound closure of porcine skin, which represents ∼10-fold extension of the tissue area achieved with conventional PTB. The results point to a new direction in photomedicine for using light in deep tissues.

## Abstract (web summary)

Light-based therapies are of growing importance in medicine, though penetrating tissue and reaching the targeted area can be difficult. Here, the authors report the use of biodegradable waveguides capable of directing light where desired, and demonstrate the potential for wound healing.

## Graphical abstract

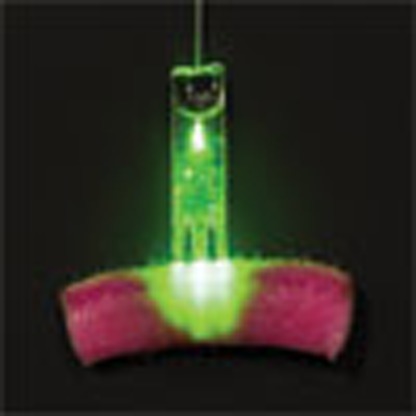

Absorption and scattering of light in tissue results from fundamental light–matter interactions^1^ and have enabled a variety of powerful optical techniques for therapy and imaging^1^,^2^,^3^,^4^,^5^,^6^,^7^,^8^,^9^. However, these interactions are also problematic as they limit the penetration of light in tissues. The 1/e penetration depth is only 1–2 mm at most for visible light^10^, and even by using near-infrared light or upconversion nanoparticles^11^,^12^ one can only increase the 1/e depth to ∼3 mm. Overcoming the limited penetration has been a challenge in almost all applications of light in photomedicine. Traditional optical fibres and endoscopes can bring light close to the surface of the target organ^13^,^14^,^15^,^16^, but delivering the light further to the target region within the tissue remains a problem.

Several biomaterials have previously been considered to form implantable photonic devices. Silk fibres^17^,^18^ have been used to fabricate patterned films^19^, diffraction gratings^20^, printed waveguides^21^, laser substrates^22^ and implantable reflectors^23^. Light-guiding hydrogel waveguides have been developed for sensing and therapies^24^,^25^,^26^,^27^,^28^. Synthetic polymers, such as poly(D,L-lactide-co-glycolide) (PLGA) and poly(L-lactic acid) (PLA), are widely used for conventional implantable devices and injectable products, for example, for resorbable sutures, but have not been explored for optical applications. These synthetic polymers exhibit advantageous properties for implantable optics, although so far they have mostly been used in an opaque form. However, these polymers can be made transparent and then have a typical refractive index of ∼1.47, adequate for guiding and delivering light in tissue. (The refractive index of the human skin is 1.38–1.44 (ref. 29).) The mechanical rigidity and flexibility of polymers can be tuned by varying their molecular weight and the degree of crosslinking. Moreover, polymers offer a wide range of biodegradation half-times, from 1 min to over a year^30^. Lactide and glycolide-based copolymers are degraded by ester hydrolysis and break into monomers, which are further degraded and resorbed in the body with minimal systemic toxicity. Therefore, we reason that these polymers are attractive candidates for making resorbable optical devices.

Here we propose a method of delivering light into living systems using bioabsorbable photonic waveguides for photomedicine. We describe the design, fabrication and characterization of waveguides made from several different transparent polymers with different mechanical and degradation properties. Further, we demonstrate an application to photochemical tissue bonding, for which a thin flexible waveguide comb is inserted into a skin incision model to deliver light uniformly along the full thickness (>10 mm) of the skin tissue. This allows watertight crosslinking between the tissues to be formed *in situ*; the waveguide need not be removed from the site after the procedure, as it is eventually biodegraded and absorbed by the tissue. This paradigm-shifting approach is expected to impact a variety of applications in the field of photomedicine, such as health monitoring, controlled drug release and chronic photodynamic therapy.

## Results

### Biopolymer waveguides

**Figure 1:**
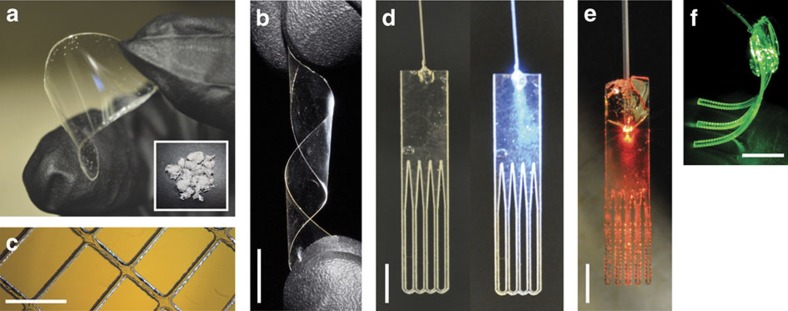
Various biopolymer films and planar waveguide demonstrations. (**a**) Transparent and flexible poly(L-lactic acid) biopolymer film obtained by melt-pressing technique. Inset: biopolymers in their initial powder form before film formation. (**b**) A twisted bio-film before laser cutting. (**c**) Laser cutting allows for a simple fabrication of films with well-controlled meshes. (**d**) A fibre-coupled waveguide array with an unstructured top region uniformly coupling the light to the array of thin waveguides. Left, off state; right, light on. Only the lower part of the devices is to be embedded into tissue, the top structure including the pigtail fibre is cut off and removed after use. (**e**) Red light coupled to a waveguide array. (**f**) A polyethylene glycol hydrogel waveguide array carrying green laser light. Scale bars, 10 mm.

To make slab waveguides, we first fabricated transparent polymer films (Fig. 1a) using melt pressing, solvent casting and ultraviolet-induced crosslinking techniques (see Methods). The thickness of the films was typically in a range from 200 to 800 μm to achieve an optimal combination of mechanical rigidity/flexibility and appropriate optical extraction efficiency depending on specific applications (Fig. 1b). The fabricated films were then cut into specific shapes by high-precision laser cutting (Fig. 1c–f). A conventional silica-based jacketed multimode fibre was pigtailed to the polymer device by using epoxy glue to couple light into the device (Fig. 1d–f). In applications, the pigtail fibre remains outside the tissue and is removed after use, and the polymer device stays in the tissue during the operation and is resorbed thereafter.

**Figure 2:**
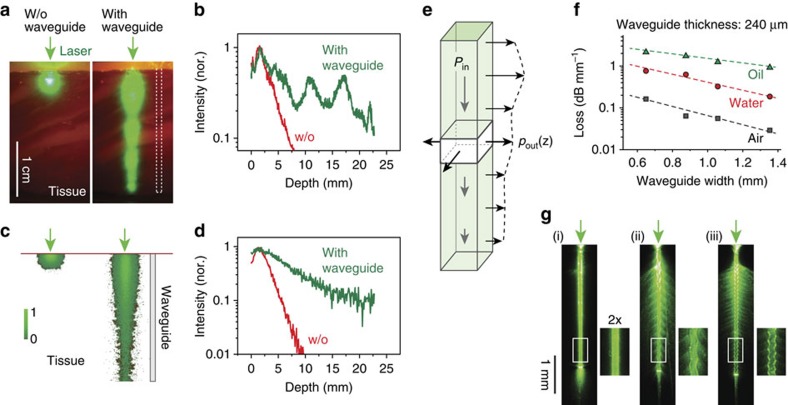
Propagation of light in biopolymer-based planar waveguides. (**a**) Microscopic image of the light in bovine tissue (thickness ∼2 mm) taken before (left panel) and after (right panel) insertion of the waveguide. Left panel: an external light source of a green laser is directly applied to the surface of the tissue. Right panel: a green laser is coupled to a waveguide implanted into the tissue. Scale bar, 10 mm. (**b**) Profile of the light decay in the tissue with (green line) and without (w/o; red line) the waveguide. (**c**) Two-dimensional ray simulations of light propagation using the same geometry as for experimental measurement in **a**. (**d**) Profile of the simulated light decay. (**e**) Schematic representation of the light extraction from a planar waveguide. *P*_out_(*z*) corresponds to the light extraction profile from the waveguide, and *P*_in_(*z*) corresponds to input per unit length. (**f**) Measured propagation loss of the waveguides in air, water and oil for increasing waveguide widths and at a constant height of 240 μm. (*N*=1 for each medium; *R*^2^ is 0.979, 0.938 and 0.906 in oil, water and air, respectively. (**g**) Waveguides with different surface patterns enable tuning the light extraction profile; here, in air. Light is coupled to the waveguide by focusing a laser to the top end of the waveguide. Scale bar, 1 mm. nor., normalized.

To investigate the waveguide-assisted light delivery in tissues, we prepared full-thickness sections of bovine skin and imaged lateral light scattering when laser light at 532 nm was delivered to just the tissue surface (epi-illumination) or launched into a waveguide embedded between two tissue sections (Fig. 2a). Without a waveguide, laser light penetrates less than 5 mm; however, with the waveguide (1 × 1 × 23 mm), bright emission was observed along the entire region of the tissue containing the waveguide (Fig. 2a). The intensity distribution along the depth indicated that the 10-dB penetration length, where the optical intensity is reduced to 10%, was extended from 7 to >23 mm (Fig. 2b). Two-dimensional ray optics simulations of our structures yielded results that matched the experimental data (Fig. 2c–d).

### Light extraction from a planar waveguide

The amount of light scattered from the waveguide as a function of depth can be easily approximated. Let *P*_out_(*z*) be the amount of light extracted from the waveguide per unit length along the depth, *z*. This output power distribution function is equal to the amount of optical loss in the waveguide (Fig. 2e), which can be expressed as:


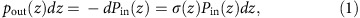


where *dz* denotes the thickness of a waveguide section, *P*_in_(*z*) is the optical power varying in *z* in the waveguide, and *σ*(*z*) is the loss coefficient of the waveguide. Note that the depth profile *P*_out_(*z*) has dimension of W m^−1^. The general solution of equation (1) is given by:


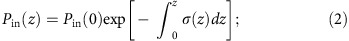



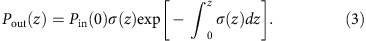


Here *P*_in_(0) is the initial power at *z*=0. The above equations describe how the intensity profiles inside and outside the waveguide are determined from the loss function *σ*(*z*). As an inverse problem, an arbitrary axial intensity distribution, *P*_out_(*z*), can be obtained in principle if the waveguide provides the (non-negative) z-dependent loss coefficient


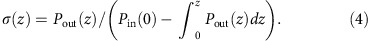


A physical interpretation of this equation is that the loss coefficient at *z* is equivalent to a ratio of the extracted power to the total power in the waveguide. As an example, for uniform power distribution in tissue over a depth of *L*, one may wish to have *P*_out_(*z*)=*a*, where *a* is a constant and *z*=[0, *L*]. From equation (3), this requires *σ*(*z*)=*a*/(*P*_in_(0)−*az*) and *P*_in_(0)≥*aL*.

In practice, the specific loss function of a waveguide can be controlled by design, such as the shape and structure, choice of material and surface quality. One of the most convenient tuning parameters is the cross-section of the waveguide. For smaller cross-sections, optical rays propagating in the waveguide bounce off the polymer/tissue interface more frequently, thus experiencing higher optical loss and, therefore, more rapid extraction into the tissue. To test this, we fabricated PLA slab waveguides from a 240-μm-thick substrate but with different waveguide widths, and measured the propagation loss by a cutback technique. To investigate the effect of different refractive index (*n*) of the surrounding, we tested waveguides in three different environments: in air (*n*=1), water (*n*=1.33) and oil (*n*=1.5), respectively. Our measurements showed that in all cases, the optical intensity in the waveguide decreased exponentially with the propagation distance, as expected from equation (2), and the loss coefficients decreased linearly with the waveguide width (Fig. 2f). For a width of 650 μm, the loss coefficient was 0.16 dB mm^−1^ or *σ*=0.37 mm^−1^ in air, but it increases to 0.76 dB mm^−1^ in water and 2 dB mm^−1^ in oil (Fig. 2f). Most soft tissues^31^ have a refractive index of 1.33–1.51. Therefore, the optical loss *in vivo* would fall somewhere between that of water and oil. A loss of 1 dB mm^−1^ may be adequate for an application where the majority (90%) of input optical energy is to be delivered through a tissue section over a depth of 10 mm.

To illuminate over thicker tissues, waveguides with lower loss would be necessary. One way to decrease the propagation loss is to increase the width; for 1.35 mm the loss coefficient in oil is 1.3 dB mm^−1^, rather than 2 dB mm^−1^ for the 650 μm discussed above (Fig. 2f). Increasing the thickness of the waveguide also reduces the loss. We fabricated PLA slab waveguides with a thickness of 440 μm and obtained consistently lower loss than for the 240-μm-thick waveguides. For example, for a width of 580 μm, the loss coefficients were 0.15 dB mm^−1^ in air, 0.62 dB mm^−1^ in water and 1.5 dB mm^−1^ in oil.

Another practical method to control the loss function is modulation of the sidewall patterns by introducing bending losses on straight waveguides. We fabricated three PLA waveguides with flat and sine-wave sidewall patterns with different periods (Λ=0.23 mm and 0.11 mm, respectively). Images of the light scattering patterns for the surface structures show drastically modified attenuation profile of the guided light and the axial profile of the extracted light intensity owing to the enhanced field deformation and curvature losses (Fig. 2g).

### Biodegradation of polymer optical waveguides

**Figure 3:**
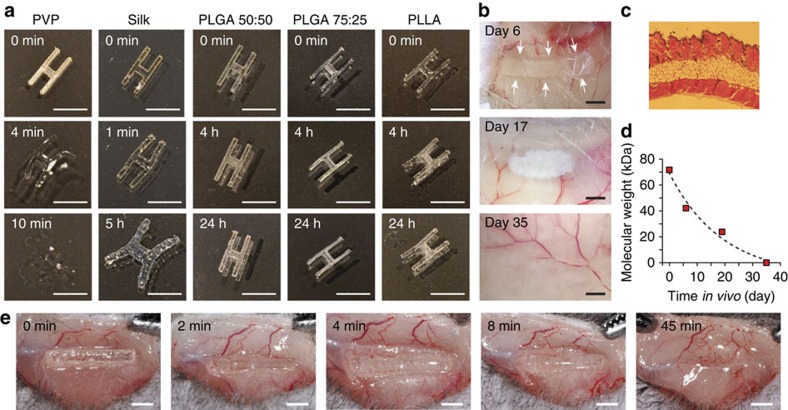
Biodegradation of biopolymer-based waveguides. (**a**) Short-term behaviour of bio-polymer films are shown by immersing H-shaped waveguide samples into PBS. PVP dissolves within minutes. Silk deforms within minutes and shows considerable swelling after a few hours. PLGA and PLA largely retain their physical structure for more than 24 h. Scale bars, 5 mm. (**b**) Time-dependent *in vivo* degradation of 1 × 5 mm PLGA 50:50 polymer subcutaneously inserted into a mouse after 6, 17 and 35 days. No inflammation around the waveguide is observed. Scale bar, 1 mm. (**c**) Histology image of skin tissue in the region where the polymer was implanted. (**d**) Average molecular weight of polymer chains in implanted bio-film as a function of degradation time (*N*=1). Line, fit to exponential decay (*R*^2^=0.93). (**e**) Dissolution of PVP waveguide *in vivo*.

We investigated the optical and mechanical properties of various polymers in an H-shaped waveguide over 24 h when immersed into phosphate-buffered solution (PBS) (Fig. 3a). Poly(vinylpyrrolidinone) (PVP), a water-soluble polymer, dissolves in PBS within minutes. Silk deforms after several minutes and swells within hours but does not fully dissolve. PLGA and PLA do not change shape in 24 h although transparency degrades modestly over the course of several hours.

To assess biodegradation of bulk polymer waveguides *in vivo*, we embedded transparent pieces of PLGA 50:50 pieces (1 × 5 mm × 500 μm) subcutaneously in live mice. The gross appearance of the implants and the surrounding tissue were examined at different time points after implantation. On day 6, we found the shape and transparency of the implant largely intact, but on Day 17, significant erosion of shape and transparency was apparent with significant loss in material volume (Fig. 3b). Visual inspection and histology indicated no sign of inflammation at the implantation site (Fig. 3c). On day 35, the implant was invisible to the naked eye. Molecular weight measurements by gel permeation chromatography showed that the molecular weight of the material decreased exponentially over time, which indicates the cleavage of polymer bonds by hydrolysis (Fig. 3d). The relative amount of GA in PLGA increases the biodegradation rate. We also tested the biodegradation of PVP and found that subcutaneously implanted PVP dissolved within 1 h *in vivo* (Fig. 3e). These results show that the biodegradability of bulk polymer waveguides varies in a large range and may be optimized for specific application by choosing appropriate polymers.

### Light delivery to deep tissue for photochemical tissue bonding

To demonstrate the effectiveness of light-delivering waveguides in medicine, we investigated PTB, which is a dye-assisted photochemical technique that induces crosslinking between wound surfaces. Among several applications of PTB, we tested skin wound enclosure. In this technique, Rose Bengal dye is applied to the wound and excited by green light to generate reactive species that induce covalent crosslinking between the collagen molecules on both sides of the tissues in the wound^32^.

**Figure 4:**
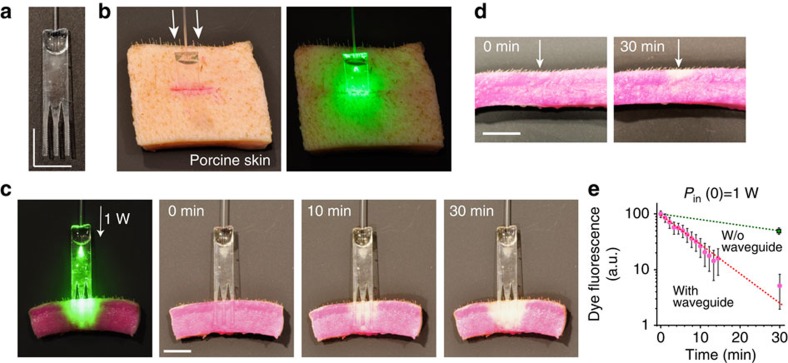
Light delivery to deep tissue. (**a**) Hybrid biopolymer-based waveguide bundle engineered for optimal light delivery to the tissue. (**b**) Waveguide inserted in porcine skin incision previously stained with Rose Bengal dye (left) and illuminated through a fibre by a green laser (right). (**c**) Cross-section of the photoactivated area of dyed porcine skin after different times of illumination using a biopolymer-based waveguide bundle. The presence of photobleaching down the entire depth of the tissue interface after 30 min of illumination indicates efficient light delivery throughout the entire depth of the sample. (**d**) Identical porcine skin sample treated with surface illumination (that is, without (w/o) using a waveguide). Note that even after 30 min of continuous illumination, only the dye close to the top surface has bleached. (**e**) The amount of remaining intact dye measured by red fluorescence (averaged over whole area of the waveguide, *N*=1) decreases exponentially with time. The error bars represent s.d. across the area. Scale bars, 10 mm in **a**,**c** and **d**.

To increase the depth to which PTB is effective in the tissue and achieve full-thickness skin bonding, we fabricated a PLA device with three waveguides. Light in the conventional multimode fibre is directly coupled to the slab waveguide with an efficiency of ∼50%, measured by a cutback technique. Each waveguide was tapered and the uniform region was 1 mm in width, 440 μm in thickness and 10 mm in length and has corrugated edges for optimal extraction of optical energy over the entire length of 10 mm (Fig. 4a). The number of waveguides can be adjusted to the size of the wound. The interspacing between the waveguides was 2.2 mm, slightly larger than the intrinsic optical penetration depth of the skin, which was optimized for efficient PTB in the inter-waveguide tissue space. We note that the thickness is comparable to the diameters (200–300 μm) of most typical absorbable sutures. For PTB, a full-thickness incision was made in the middle of the porcine skin, and the comb waveguides were inserted into the incision (Fig. 4b).

Before the tissue-bonding experiment, we cut a thick piece of porcine skin tissue into two halves. After applying Rose Bengal dye onto the exposed surface of one half, a waveguide was placed and 532-nm laser light at 1 W was launched to the pigtail fibre. The optical image shows a uniform distribution of optical energy in the tissue around the waveguides (Fig. 4c). The optical intensity at the tissue interface over an area of ∼1 × 1 cm^2^ is therefore 1 W cm^−2^, which complies with the maximum permissible exposure (1–4 W cm^−2^) of skin *in vivo* to prolonged laser irradiation at 400–700 nm (ref. 33). To measure the time-lapse photoactivation of the dye, we imaged the cross-section at various time points after laser irradiation. The two pieces of tissue were brought into contact during the light delivery to simulate the actual situation of PTB. The time-lapse images of the tissue cross-section showed photobleaching of the dye in the superficial area of the skin after 10 min, but almost complete bleaching occurred over a large area around the entire implant after 30 min (Fig. 4c). Photobleaching results from degradation of the dye over multiple excitations. We note that complete photobleaching is not always required for effective PTB.

For comparison, we illuminated the surface of a piece of porcine skin tissue directly with an unfocused 532-nm laser beam with a diameter of 3 mm at various power levels. We found that photobleaching was limited to a superficial layer at depths of less than 1.5 mm even at a high power of 1 W (14 W cm^−2^; Fig. 4d). The total amount of fluorescence from unbleached dye was measured from the region of interest in direct contact with the waveguide. The results indicated an exponential increase of photobleaching over time (Fig. 4e).

**Figure 5:**
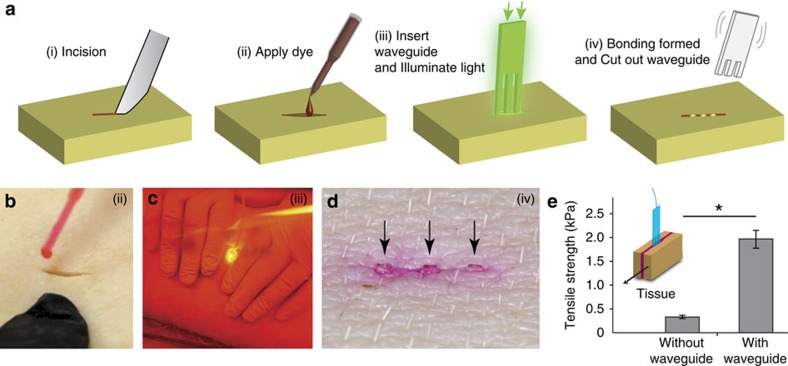
Waveguide-assisted photochemical tissue bonding. (**a**) Schematic of the experimental procedure. (i) A full-thickness skin incision is performed. (ii) Rose Bengal dye is applied to the surfaces of a wound in porcine skin, and the excess solution is removed. (iii) A waveguide is inserted into the wound, and light is delivered to the waveguide through an optical fibre. (iv) After the procedure, the waveguide is trimmed away close to the skin surface. (**b**–**d**) Photographs showing steps (ii), (iii) and (iv). The arrows in **d** indicate the position of the waveguide inside the tissue. (**e**) Shear tensile strength of PTB bonds formed *ex vivo* in porcine skin. PTB bonds formed using a waveguide show more than fivefold increased bonding strength compared with conventional superficial illumination of the skin. *P value <0.01 in *t*-test (*N*=3). The errors bars represent the s.d.

Next, we tested waveguide-assisted PTB *in situ* in animals (Fig. 5a). A full-thickness incision was made on the dorsal skin of a pig immediately after being killed (Fig. 5b). After applying Rose Bengal dye to the wound, a polymer waveguide was inserted into the wound. The tissue was approximated to ensure physical contact between tissues and waveguide surface while 532-nm laser light at 1 W was delivered over a duration of 15 min (Fig. 5c). After the irradiation was completed, the exposed part of the waveguide was cut away, leaving the remaining implant within the treated tissue (Fig. 5d). If conventional non-biodegradable fibres had been used, they would have to be removed from the tissue, and this would damage the treated tissue and disrupt the wound closure. Biodegradable polymers, however, will be gradually resorbed and replaced by native tissues if used in an *in vivo* setting. To measure the bond strength between the pieces of tissue, we performed tensometer-based shear tensile measurements (Fig. 5e). Conventional PTB without a waveguide yielded a tensile strength of 0.33 kPa. By contrast, waveguide-assisted PTB showed a much higher strength of 1.94 kPa due to full-thickness photoactivation of porcine skin.

## Discussion

To date, biocompatible and biodegradable polymers have been extensively studied and used in many fields of medicine such as cardiovascular medicine^34^, orthopedics^35^ and neurology^36^. They show minimal systemic toxicity and the rate of resorption of polymeric implants can be tailored to minimize the probability of tissue irritation during degradation. Therefore, they provide a safe material platform to be used for new device applications.

PTB offers a number of advantageous features such as the formation of a water-tight closure across the entire wound interface and minimal scar formation^32^,^37^, compared with wound closure by standard sutures and staples or by cyanoacrylate and fibrin glues. However, until today^32^,^38^, the application of PTB has been limited to superficial wounds with a maximum depth of 1–2 mm. By using biodegradable waveguides, the remit of PTB is substantially expanded and no longer limited in terms of the size of the wound or the type and transparency of the tissue. Moreover, it is known that the efficiency of photochemical PTB is sensitive to oxygen concentration and greatly enhanced in live animals compared with the *ex vivo* tissues used here^39^. We therefore expect a considerably higher tensile strength for *in vivo* full-thickness PTB.

The potential applications of biodegradable polymer waveguides are expected to go far beyond PTB. Implantable waveguides can extend the therapeutic depths in photodynamic and photothermal therapies, enable deep tissue stimulation by low-level light or optogenetic techniques, and offer new strategies for continuous monitoring of medical conditions and sensing diagnostic information. Furthermore, biodegradable graded index lenses or fibres may permit longitudinal imaging of the viability of transplanted organs and monitoring of the healing process after surgery.

In conclusion, we have developed a new class of biocompatible and biodegradable polymer waveguides and validated their effectiveness for inducing photochemical processes in deep tissue. A comb-shaped slab waveguide that was designed and fabricated for optimal light delivery enabled us to demonstrate successful PTB treatment of a full-thickness skin incision (>1 cm deep), which has not been possible by conventional surface illumination and would be impractical with non-biodegradable optical materials. Although the initial focus of this study was on light-delivering devices for PTB, biocompatible and biodegradable waveguides can be directly applied to other light-based diagnostics, surgery and therapeutics. Therefore, we expect that bioabsorbable waveguides may initiate a new paradigm and find widespread use in medicine.

## Methods

### Polymer waveguide fabrication

We purchased poly(D,L-lactide-co-glycolide) (Sigma, acid-terminated lactide:glycolide 50:50 with a MW of 38,000–54,000 Da or ester-terminated lactide:glycolide 75:25 with a MW of 76,000–115,000 Da) and poly(lactic acid) (Hycail CML-PLA, MW of 63,000±12,000 Da) as dry powders. The powders were placed onto a glass slide preheated to 230 °C on a hot plate. On melting, another glass slide was used to press the polymer to a thin film. The glass slides were treated beforehand with Sigmacote (Sigma) to prevent sticking of the polymer to the glass. Glass spacers were used to precisely control the thickness of the films. To cool and separate the film from the glass slides it was immediately submerged into icy water. The poly(vinylpyrrolidinone) (PVP) (Sigma, MW of 360,000 Da) films were prepared by solvent evaporation. PVP was dissolved in water at a concentration of 20 wt%, was poured into a petri dish and dried in air to make the PVP films. Polyethylene glycol hydrogel films were prepared by photopolymerization of the precursor solution containing 80 wt% polyethylene glycol diacrylate (Sigma, MW of 700 Da), 5 wt% 2-hydroxy-2-methylpropiophenone (Sigma) and 15 wt% water using a ultraviolet lamp. Thin films were cut to the desired shape using VersaLaser VLS3.50 cutting/engraving system at 5 mm s^−1^ with a power of 200 mW. To make silk waveguides, the extraction of silk fibroin was performed as described elsewhere^40^. The extracted silk fibroin was concentrated to 20% by dialysis. This solution was dried in waveguide-shaped mould overnight. The waveguides from all the above materials were bonded to a standard multimode optical fibre (200-μm core, 0.48 NA) using an optical adhesive (Norland, NOA 81).

### Measurement of waveguide loss

We used the cutback technique to measure the propagation losses of waveguides in air, water and oil. Light from a 635-nm diode laser (Coherent) or a 532-nm miniature diode-pumped Nd:YAG laser (Laser Quantum) is coupled via an objective lens to the waveguide and the light power at the output of the waveguide is measured with a power meter at various lengths. Finally, the propagation loss is calculated according to the measured power levels at different lengths.

### Light propagation simulation

The light propagation in waveguides embedded in skin tissue was simulated by using the TracePro ray-tracing software. A light source in the waveguide (that is, influx surface) has a boundary shape of annular and a grid pattern of circular that provide ∼10^5^ rays for tracing. The waveguide has a refractive index of 1.476 and the side surface of the waveguides is modelled with a diffuse reflection of 0.92. The tissue has an anisotropy of 0.81, absorption coefficient of 0.145 mm^−1^ and scattering coefficient of 1.5 mm^-1^. The waveguide in the tissue has dimensions of 1, 0.44 mm and 2 cm. The tissue has a total thickness of 2 mm. The light on the tissue surface is observed 3 mm above it.

### Implantation

We used immunocompetent male C57BL6 mice, ages 8–16 weeks, for the implantation study. After anesthesia by intraperitoneal injection of ketamine (100 mg kg^−1^) and xylazine (10 mg kg^−1^), the dorsal skin was incised over ∼1 cm longitudinally and the implant was inserted subcutaneously. The incision was then closed using a 6–0 nylon suture. All animal experiments were performed in compliance with institutional guidelines and approved by the subcommittee on research animal care at the Harvard Medical School.

### Histology

A sample of full-thickness skin around the implant was excised and fixed in 4% formalin for over 48 h. The skin sample was frozen-sectioned with a microtome at a thickness of 5 μm and stained with haematoxylin and eosin. The slide was imaged with a bright-field microscope (Olympus).

### Photobleaching and PTB experiments

Photobleaching and PTB experiments are done on porcine tissue. The tissues are harvested immediately after the animals were killed and stored at −20 °C. Immediately before use, the tissues were defrosted in a water bath for 20 min. The waveguide-assisted PTB procedure (in Fig. 5a–c) was demonstrated on a pig immediately after the animal was killed. An incision was made in the tissue and a solution of 0.1% (w/v) Rose Bengal (Aldrich) in PBS was applied to each exposed surface of the opened wound. After 1 min, the excess dye solution was wiped off. The tissue surfaces covered by Rose Bengal were illuminated by a 532-nm continuous-wave KTP-frequency-doubled solid-state laser (LRS-0532-PFH-000500-05, Laserglow Technologies, Canada, 800 mW, 532 nm), at which the Rose Bengal has an extinction coefficient of 25,000 M^−1^ cm^−1^ in PBS. The bonding strength of attached tissues was measured by shear tensile technique using the tensiometer (MTESTQuattro, Admet). The *P* value of the bonding strengths with and without waveguides is 0.0017 in *t*-test.

## Additional information

**How to cite this article:** Nizamoglu, S. *et al.* Bioabsorbable polymer optical waveguides for deep-tissue photomedicine. *Nat. Commun.* 7:10374 doi: 10.1038/ncomms10374 (2016).
